# Association of White Blood Cell Count and Differential with the Incidence of Atrial Fibrillation: The Atherosclerosis Risk in Communities (ARIC) Study

**DOI:** 10.1371/journal.pone.0136219

**Published:** 2015-08-27

**Authors:** Jeffrey R. Misialek, Wobo Bekwelem, Lin Y. Chen, Laura R. Loehr, Sunil K. Agarwal, Elsayed Z. Soliman, Faye L. Norby, Alvaro Alonso

**Affiliations:** 1 Division of Epidemiology and Community Health, School of Public Health, University of Minnesota, Minneapolis, Minnesota, United States of America; 2 Cardiovascular Division, Department of Medicine, University of Minnesota Medical School, Minneapolis, Minnesota, United States of America; 3 Department of Epidemiology, UNC Gillings School of Global Public Health, University of North Carolina, Chapel Hill, North Carolina, United States of America; 4 Division of Medicine, John Hopkins University School of Medicine, Baltimore, Maryland, United States of America; 5 Department of Epidemiology, John Hopkins Bloomberg School of Public Health, Baltimore, Maryland, United States of America; 6 Epidemiological Cardiology Research Center (EPICARE), Wake Forest School of Medicine, Winston-Salem, North Carolina, United States of America; 7 Department of Epidemiology and Prevention, Wake Forest School of Medicine, Winston-Salem, North Carolina, United States of America; 8 Department of Internal Medicine (Cardiology Section), Wake Forest School of Medicine, Winston-Salem, North Carolina, United States of America; University of Louisville, UNITED STATES

## Abstract

**Background:**

Although inflammation is involved in the development of atrial fibrillation (AF), the association of white blood cell (WBC) count and differential with AF has not been thoroughly examined in large cohorts with extended follow-up.

**Methods:**

We studied 14,500 men and women (25% blacks, 55% women, mean age 54) free of AF at baseline (1987–89) from the Atherosclerosis Risk in Communities (ARIC) study, a community-based cohort in the United States. Incident AF cases through 2010 were identified from study electrocardiograms, hospital discharge records and death certificates. Multivariable Cox proportional hazards regression was used to estimate hazard ratios (HR) and 95% confidence intervals (CI) for AF associated with WBC count and differential.

**Results:**

Over a median follow-up time of 21.5 years for the entire cohort, 1928 participants had incident AF. Higher total WBC count was associated with higher AF risk independent of AF risk factors and potential confounders (HR 1.09, 95% CI 1.04–1.15 per 1-standard deviation [SD] increase). Higher neutrophil and monocyte counts were positively associated with AF risk, while an inverse association was identified between lymphocyte count and AF (multivariable adjusted HRs 1.16, 95% CI 1.09–1.23; 1.05, 95% CI 1.00–1.11; 0.91, 95% CI 0.86–0.97 per 1-SD, respectively). No significant association was identified between eosinophils or basophils and AF.

**Conclusions:**

High total WBC, neutrophil, and monocyte counts were each associated with higher AF risk while lymphocyte count was inversely associated with AF risk. Systemic inflammation may underlie this association and requires further investigation for strategies to prevent AF.

## Introduction

Atrial fibrillation (AF) is a growing health concern in the United States. Currently, over two million individuals have AF, and its prevalence is anticipated to double by 2050.[1] In addition to being the most frequently observed sustained cardiac arrhythmia in clinical practice, AF has been linked with increased risk of cardiovascular disease (CVD), heart failure (HF), myocardial infarction (MI), stroke, and overall mortality.[2–5] However, a considerable proportion of the attributable risk of AF (44%) remains unexplained after accounting for one or more borderline or elevated risk factors.[6]

Higher levels of systemic inflammation, usually determined with C-reactive protein, have been associated with AF risk.[7,8] White blood cell (WBC) count can also be considered a biomarker of inflammation and potentially related to an increased risk of AF. In selected patient samples, an elevated WBC count during the perioperative period was identified as being predictive of postoperative AF[9–12] and with recurrent AF after pulmonary vein isolation.[13] The association between WBC count and AF in the general population has been examined previously in a subset of the Framingham Heart Study and a study in Norway. Among 936 eligible participants followed up for a maximum of 5 years in the Framingham Heart Study, higher WBC count was associated with increased risk of AF. However, this association was no longer significant after additional follow up.[14] In Norway, the Tromsø study also identified a positive association between total WBC count and AF risk, while there was no association between WBC differential with AF risk.[15] However, granulocyte count (especially neutrophils) has been linked to increased incidence of other CVD,[16,17] and evidence suggests that myeloperoxidase (MPO), an enzyme abundantly produced by neutrophils, may be involved in the development of atrial fibrosis, resulting in an increased risk of AF.[18,19] Therefore, studying the association of WBC differential counts with AF in a larger sample size and in a racially diverse population might clarify the association of WBC and its differential with AF.

We prospectively examined the relationship between total WBC count with incident AF in the Atherosclerosis Risk in Communities (ARIC) Study, a community-based cohort that included a large sample of middle-aged participants, followed for an extended period. Also, we explored the association between each component of WBC differential and AF incidence separately. Overall, we hypothesized that individuals with higher WBC count would have an increased risk for AF, and this association was due to an increased risk of AF associated with higher levels of granulocytes, mainly neutrophils.

## Methods

### Study Design and Subjects

The ARIC study is a prospective, community-based cohort that aimed to explore risk factors of atherosclerosis and CVD.[20] Briefly, a total of 15,792 men and women aged 45–64 years located in four communities from around the United States (Forsyth County, North Carolina; Jackson, Mississippi; the northwest suburbs of Minneapolis, Minnesota; and Washington County, Maryland) were recruited at the baseline examination in 1987–1989 (visit 1), which included a home interview and a clinical visit. Additional study examinations occurred in 1990–1992 (visit 2), 1993–1995 (visit 3), 1996–1998 (visit 4), and 2011–2013 (visit 5). This cohort consists of African Americans from Jackson, mostly whites from Minneapolis and Washington County, and both African Americans and whites from Forsyth County. The University of Minnesota Institutional Review Board approved the present study, and all participants enlisted in the study have given their written informed consent.

**Fig 1:**
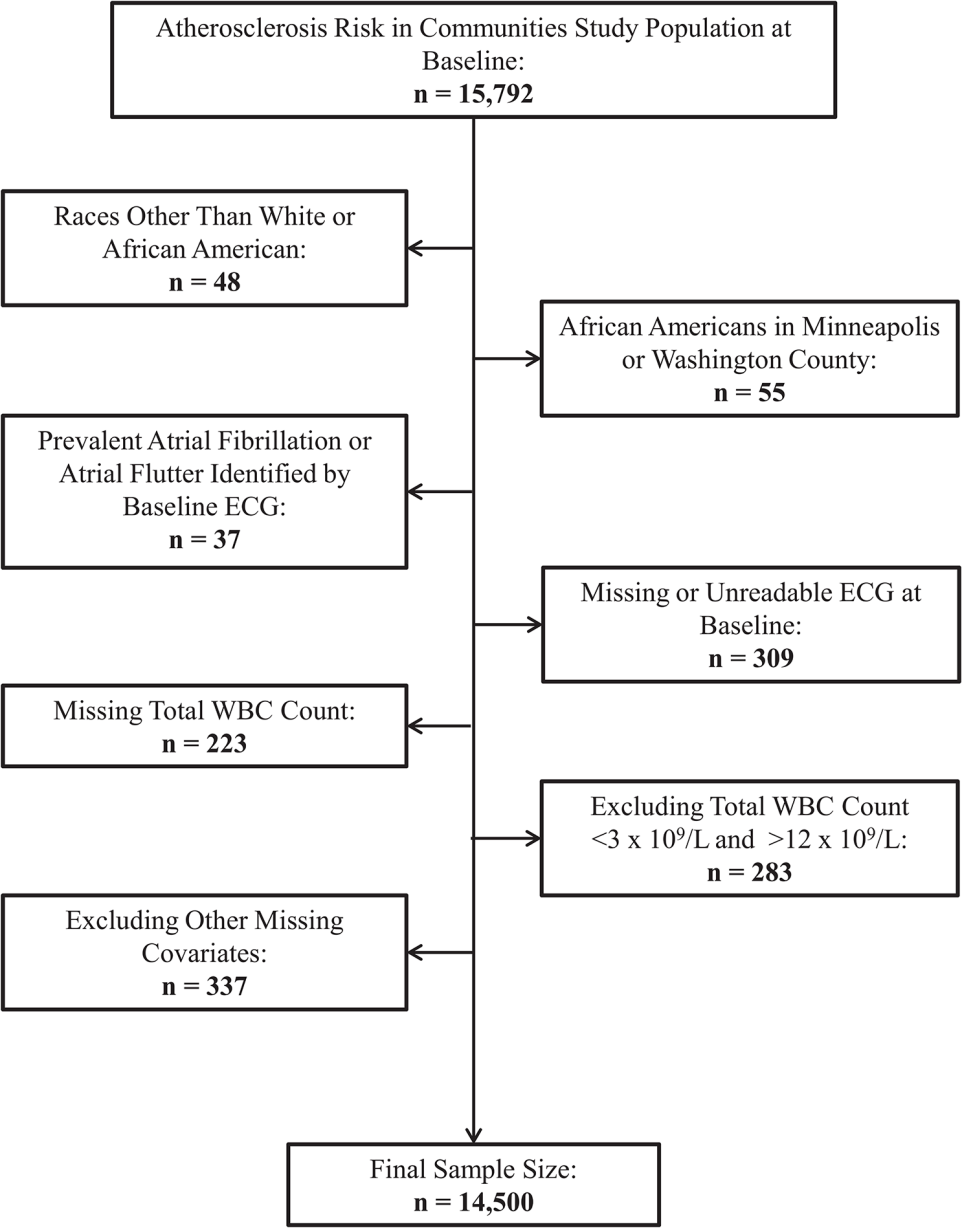
Flow chart of participants excluded at baseline, Atherosclerosis Risk in Communities Study, 1987 to 1989. ECG = electrocardiogram. WBC = White Blood Cell.

From the initial ARIC sample of 15,792 individuals, the following participants were excluded: those with races other than white or African-American due to small sample sizes (n = 48); African Americans who were located in either Minneapolis or Washington County due to small sample sizes (n = 55); those with prevalent AF or atrial flutter diagnosed by electrocardiograms (ECG) at baseline (n = 37); those with a missing or an unreadable baseline ECG (n = 309); those who were missing baseline WBC count measurements (n = 223); those with total WBC counts less than 3 x 10^9^/L (one percentile) or greater than 12 x 10^9^/L (99^th^ percentile) due to a concern for other occult diseases that may affect WBC count (n = 283); and those who were missing any information on other covariates (n = 337). Overall, the final analytic samples consisted of 14,500 participants for the total WBC analyses (Fig 1). The WBC differential analyses have a reduced sample size (n = 10,661) since individual WBC differential measurements were not available for all ARIC participants.

### Assessment of Total WBC Count/Differential and Other Covariates

Fasting blood samples were drawn from the antecubital vein, and plasma and serum were frozen at -70°C until they were analyzed. WBCs were retrieved from whole anti-coagulated blood, and WBC count was determined by automated particle Coulter Counters within 24 hours after venipuncture in local hospital hematology laboratories. Total WBC count was determined in the whole blood sample. The reliability coefficient for the WBC count measurement was greater than 0.96.[21] Fibrinogen plasma levels were determined in the ARIC Central Hemostasis Laboratory using the thrombin time titration method with reagents obtained from General Diagnostics Organon Technica Co. (Morris Plains, New Jersey) from previously established procedures.[22] Measurements repeated on a sample of participants over several weeks yielded reliability coefficients of 0.72 for fibrinogen.[23] Serum albumin was measured with a Coulter DACOS instrument (Coulter Diagnostics, Hialeah, Florida) with a bromcresol green colorimetric assay.[24] The reliability coefficient of albumin measurements based on repeated testing of 40 healthy participants over four weeks was 0.69, and the within-person variability was 2.8 percent.[25]

Questionnaires were used at each study exam to assess self-reported information such as smoking status, pack-years, drinking status, education level, and medication usage. Pack-years were calculated by the average number of cigarettes per day divided by 20 cigarettes per pack and then multiplied by the number of years smoked. In addition, all medications including antihypertensives used in the preceding 2 weeks before each clinic visit were documented. Seated blood pressure was measured after five minutes of rest using a random-zero sphygmomanometer, and systolic blood pressure was defined as the average of the last 2 of 3 consecutive measurements. Height and weight were measured while the individual was wearing a scrub suit and no shoes, and body mass index (BMI) was defined as weight in kilograms divided by height in meters squared. Chronic obstructive pulmonary disease (COPD) was determined through a self-reported physician diagnosis of chronic bronchitis or emphysema. Prevalent diabetes mellitus was characterized by a fasting glucose ≥126 mg/dL, non-fasting glucose ≥200 mg/dL, a self-reported physician diagnosis, or use of anti-diabetic medications. Prevalent MI at baseline was characterized by a previously reported MI event or from ECG while prevalent HF was defined by the intake of HF medications or a score of 3 on the Gothenburg criteria.[26] Prevalent stroke was designated by a self-reported history of physician-diagnosed stroke. Incident cases of HF, MI, or stroke were identified through study exams, annual telephone interviews, and the ongoing surveillance of ARIC community hospitals for any hospitalizations or deaths of any cohort participants.

### Incident AF events

AF cases within the ARIC cohort have been ascertained from three sources: ECGs at each of the five study exams, diagnostic codes from hospitalization discharge summaries, and death certificates.[27,28] A 12-lead, 10-second ECG during each study visit was performed with each participant lying in a supine position. After the ECGs were transmitted electronically to the ARIC ECG Reading Center (EPICARE, Wake Forest University Health Sciences, Winston-Salem, North Carolina), they were reviewed for technical quality and were electronically processed using 2001 version of the GE Marquette 12-SL program (GE Healthcare, Milwaukee, Wisconsin). A trained cardiologist visually rechecked and confirmed any ECGs that were automatically coded as an AF or atrial flutter diagnosis.[29]

Hospitalizations and deaths in the ARIC study are identified through annual follow-up phone calls to the study participants (>90% participation) and continuous surveillance of local hospital discharge lists, the National Death Index, and state registries for any cardiovascular events or deaths. Trained abstractors compile pertinent information from all participants’ hospitalizations. Using International Classification of Diseases, ninth revision, clinical modification (ICD-9-CM) codes for diagnoses and procedures associated with each hospitalization, AF was identified when 427.31 or 427.32 was listed as a discharge diagnostic code in any hospitalization. Any AF event identified during a hospitalization for open cardiac surgery was excluded. In a physician review of discharge summaries from 125 possible AF cases within the ARIC study, approximately 90% of the cases were confirmed, and the sensitivity was >80%.[27] For AF ascertainment through death certifications, incident AF cases were determined as a cause of death if ICD-9 code 427.3 or ICD-10 code I48 was listed. The vast majority of incident AF cases (>98%) in this analysis were identified from hospitalization discharge codes, while less than 1% of AF cases were identified from death certificates. AF incidence date was defined as the first date in which an AF diagnosis by ECG, hospitalization, or death occurred.

### Statistical Analysis

Baseline characteristics of participants stratified by total WBC count quintiles and then by separate WBC differential quintiles and groups are reported using means and standard deviations (SD) for continuous variables and percentages for categorical variables. For the primary analysis, Cox proportional hazards regression was used to calculate adjusted hazard ratios (HRs) and 95% confidence interval (CI) for the association of total WBC count with incident AF. The association of WBC differential count with incident AF was investigated in separate Cox regression models for each component of WBC differential. Follow-up time was defined as the time between the baseline visit and the date of AF incidence, death, loss to follow-up, or December 31, 2010, whichever came first. Death and loss to follow-up were considered to be censoring events with n = 3428 dying from causes other than AF and n = 591 being lost to follow-up.

Initially, restricted cubic splines with knots at the 5^th^, 27.5^th^, 50^th^, 72.5^th^ and 95^th^ percentiles were utilized to explore the shape of the dose-response association of total WBC count and each WBC differential with AF risk. For the Cox regression analysis, total WBC count and all WBC differential except basophils were classified as quintiles while basophils were classified as 3 groups due to the limited range. In addition, both total WBC and WBC differential were modeled as linear variables per 1-SD unit change (total WBC count SD = 1.70 x 10^9^/L, neutrophil count SD = 1.42 x 10^9^/L, lymphocyte count SD = 0.64 x 10^9^/L, neutrophil count/lymphocyte count ratio SD = 1.25, monocyte count SD = 0.18 x 10^9^/L, eosinophil count SD = 0.15 x 10^9^/L, and basophil count SD = 0.04 x 10^9^/L). The initial model was adjusted for age, race, sex, and ARIC field center site at baseline. The second model was further adjusted for other baseline variables including COPD (yes, no), diabetes (yes, no), drinking status (current, former, never), education level (some high school or less, high school graduate/vocational school, college/graduate school), smoking status (current, former, never), use of antihypertensive medications (yes, no), prevalent MI (yes, no), prevalent HF (yes, no), prevalent stroke (yes, no), and the following continuous variables: BMI (kg/m^2^), pack-years, height (cm), and systolic blood pressure (mmHg). Lastly, the third model was adjusted for incident MI, HF, and stroke as time-dependent covariates and potential mediators of the association between total WBC count/differential and AF risk since they are risk factors for AF and may be on the causal pathway.

Effect modification was evaluated by age, sex, race, BMI, and smoking conducting stratified analysis and including multiplicative terms between the effect modifier and total WBC count. The proportional hazards assumption was explored through the examination of an interaction term between each WBC variable and follow-up time along with the inspection of log-negative log survival curves; no departures from the assumption were observed. In a sensitivity analysis, individuals who had an incident AF event within the first two years of follow-up from baseline were excluded to avoid including potentially undetected prevalent AF cases as incident cases. Also, other inflammatory markers measured at baseline (fibrinogen and albumin) were included in Model 2 as an additional analysis to examine the effect of adjusting for other inflammatory markers on the total WBC count/differential and incident AF associations. Additional analyses were performed to examine the impact of the competing risk of death in the association between WBC and AF using competing-risk regression models[30] and to evaluate the association of total WBC count and differential with AF cases ascertained by ECG and hospitalization separately. Finally, an analysis restricting follow-up to the first 10 years of follow-up was done to compare the results to previous studies that had similar follow-up periods. All statistical analyses were performed using SAS v 9.2 (SAS Inc., Cary, North Carolina).

## Results

At baseline, 14,500 participants between the ages of 45 and 64 were free of AF and met the inclusion criteria for this analysis. Median (25^th^–75^th^ percentile) total WBC count was 5.8 x 10^9^/L (4.8 x 10^9^/L–7.1 x 10^9^/L) for the entire cohort. Table 1 shows the characteristics of the ARIC participants by total WBC count quintiles. A positive association was seen with several risk factors for CVD such as BMI, diabetes, smoking, antihypertensive medications, and COPD, while lower total WBC counts were seen in females and in African Americans. Baseline characteristics by quintiles of specific components of WBC differential are shown in Tables A-F in S1 File.

**Table 1:** Baseline characteristics by total white blood cell (WBC) count quintile, Atherosclerosis Risk in Communities Study, 1987 to 1989.

	Total WBC Count (x 10^9^/L)†				
	3.0–4.6	4.7–5.4	5.5–6.2	6.3–7.4	7.5–12.0
	(n = 2970)	(n = 2966)	(n = 2769)	(n = 3002)	(n = 2793)
Total WBC count median, x 10^9^/L	4.1	5.1	5.8	6.8	8.5
Age, years	53.9 (5.6)	54.1 (5.7)	54.3 (5.7)	54.4 (5.9)	54.1 (5.8)
Females, %	59.5	57.2	54.0	53.1	50.4
African Americans, %	37.4	26.8	20.9	20.3	18.4
Body mass index, kg/m^2^	26.9 (5.0)	27.4 (5.1)	27.9 (5.3)	28.2 (5.5)	28.0 (5.6)
Chronic obstructive pulmonary disease, %	5.9	7.5	9.8	11.0	13.5
Current drinker, %	51.8	56.8	58.5	56.8	59.6
Current smoker, %	10.1	14.6	18.8	30.4	55.0
Pack-years	8.6 (16.8)	11.1 (17.7)	14.4 (20.2)	19.3 (23.1)	27.3 (24.9)
Diabetes, %	7.1	8.7	11.6	13.7	17.0
Height, cm	168.4 (9.3)	168.4 (9.3)	168.7 (9.3)	168.4 (9.4)	168.7 (9.2)
High school degree, %	39.2	40.4	42.5	42.0	42.7
Systolic BP, mmHg	119.8 (18.5)	121.1 (18.6)	121.5 (18.3)	121.8 (19.1)	121.4 (19.0)
Use of antihypertensive medications, %	26.5	27.8	29.9	32.5	34.4
Prevalent heart failure, %	2.5	3.7	3.7	5.3	7.2
Prevalent myocardial infarction, %	2.3	2.6	4.4	4.5	6.3
Prevalent stroke, %	1.7	1.4	1.6	2.1	2.2
Basophil count, x 10^9^/L*	0.03 (0.03)	0.03 (0.03)	0.03 (0.04)	0.04 (0.05)	0.04 (0.06)
Eosinophil count, x 10^9^/L*	0.1 (0.1)	0.1 (0.1)	0.1 (0.1)	0.2 (0.1)	0.2 (0.2)
Lymphocyte count, x 10^9^/L*	1.6 (0.4)	1.8 (0.5)	1.9 (0.5)	2.1 (0.6)	2.5 (0.7)
Monocyte count, x 10^9^/L*	0.3 (0.1)	0.3 (0.1)	0.3 (0.1)	0.4 (0.2)	0.5 (0.2)
Neutrophil count, x 10^9^/L*	2.0 (0.6)	2.7 (0.6)	3.3 (0.6)	4.0 (0.7)	5.5 (1.2)
Neutrophil/lymphocyte ratio*	1.5 (0.7)	1.7 (1.1)	1.9 (0.9)	2.1 (1.3)	2.6 (1.7)

BP indicates blood pressure. Values are mean (SD) when appropriate.

*Reduced sample size: n = 10,661

†All baseline characteristics have p-values <0.05 for differences in means (ANOVA) and percentages (Chi-Square) between total WBC quintiles except for height and prevalent stroke.

### Total WBC Count and AF Risk

**Fig 2:**
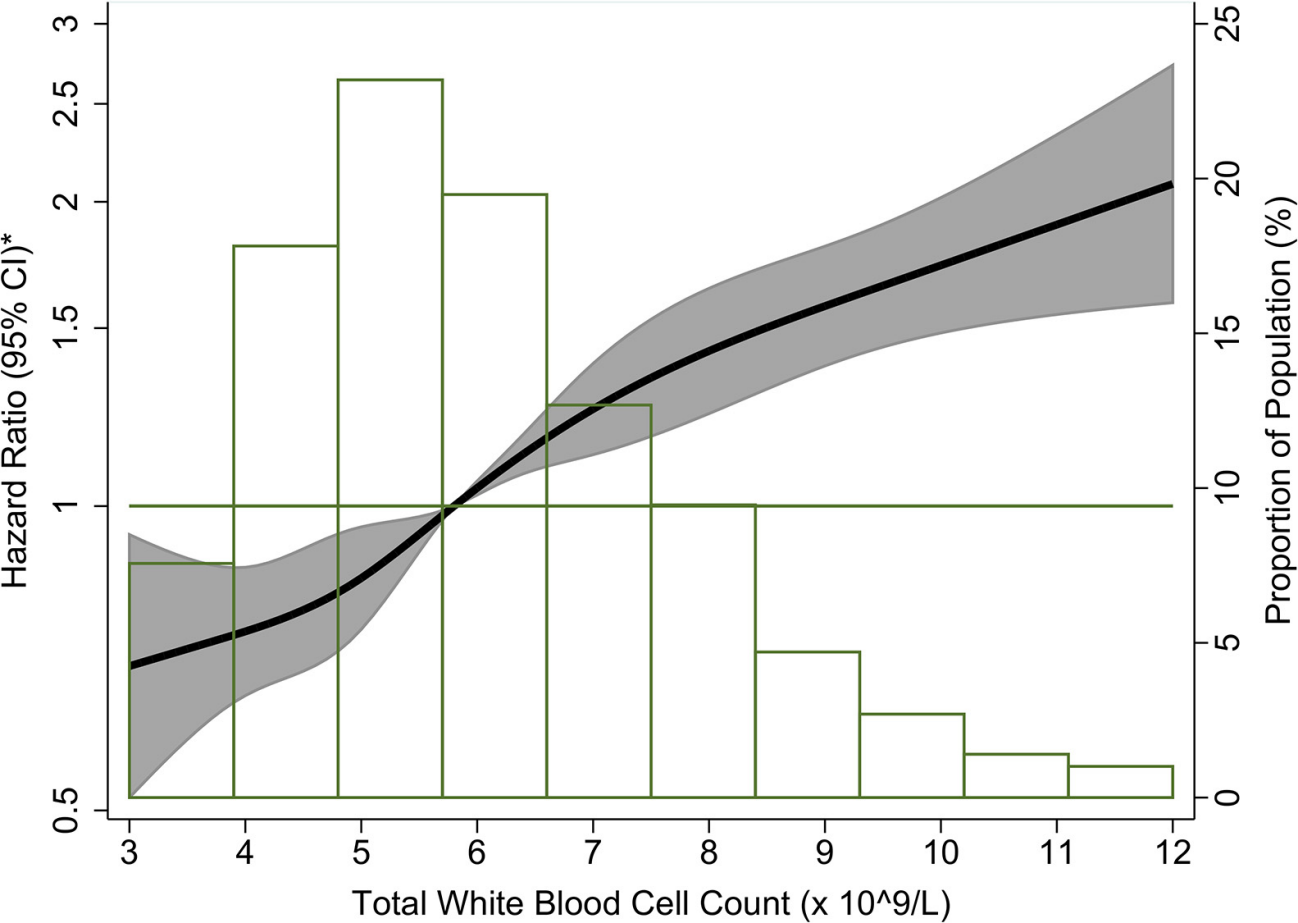
Association between total white blood cell count and incident atrial fibrillation presented as hazard ratio (solid line) and 95% confidence intervals (shaded area), Atherosclerosis Risk in Communities Study, 1987 to 2010. *Cox proportional hazards model using restricted cubic splines with knots at the 5^th^, 27.5^th^, 50^th^, 72.5^th^ and 95^th^ percentiles and adjustment for age, race, and sex. The reference is the median value of the total white blood cell count (hazard ratio = 1), and the histogram represents the frequency distribution of the total white blood cell count in the study sample.

A total of 1928 ARIC participants were classified as having an incident AF event through December 31, 2010, with a median follow-up of 21.5 years for the whole study population. After adjustment for age, race, and sex, total WBC count was positively and linearly associated with AF incidence (Fig 2). In Table 2 (model 2), participants in the highest total WBC count quintile (7.5–12.0 x 10^9^/L) had a 23% higher AF risk than those in the lowest quintile (3.0–4.6 x 10^9^/L) after adjustment for AF risk factors and potential confounders (HR: 1.23; 95% CI: 1.05–1.44). When total WBC count was modeled as a linear variable in an adjusted model, a 1-SD increase in total WBC count was associated with a 9% higher risk of incident AF (HR: 1.09; 95% CI: 1.04–1.15; Table 2, model 2). Adjustment for incident CVD as a time-varying covariate (Table 2, model 3) weakened the total WBC count-incident AF association to non-significance in both the quintile and linear results. There were no interactions by age, sex, race, BMI, and smoking.

**Table 2:** Hazard ratio (HR) and 95% confidence interval (CI) of atrial fibrillation (AF) by total white blood cell (WBC) count, Atherosclerosis Risk in Communities Study, 1987 to 2010.

		Total WBC Count (x 10^9^/L)				
	3.0–4.6	4.7–5.4	5.5–6.2	6.3–7.4	7.5–12.0	Linear
	(n = 2970)	(n = 2966)	(n = 2769)	(n = 3002)	(n = 2793)	(per 1-SD* Increase)
**AF Cases**	300	326	377	453	472	1928
**Person-years**	58,663	57,492	52,712	54,358	48,137	271,362
**Crude Incidence Rate (per 1000 person-years)**	5.1	5.7	7.2	8.3	9.8	7.1
**Model 1 HR**	1	1.08	1.33	1.59	1.97	1.28
**(95% CI)**	(Reference)	(0.92–1.26)	(1.14–1.55)	(1.37–1.84)	(1.70–2.28)	(1.23–1.34)
**Model 2 HR**	1	0.99	1.08	1.16	1.23	1.09
**(95% CI)**	(Reference)	(0.84–1.16)	(0.93–1.26)	(1.00–1.36)	(1.05–1.44)	(1.04–1.15)
**Model 3 HR**	1	0.99	1.03	1.05	1.07	1.03
**(95% CI)**	(Reference)	(0.84–1.16)	(0.88–1.20)	(0.90–1.23)	(0.91–1.26)	(0.98–1.08)

**Model 1:** Cox proportional hazards model adjusted for age, race, sex, and study site.

**Model 2:** Model 1 with additional adjustment for body mass index, chronic obstructive pulmonary disease, diabetes mellitus, drinking status, educational level, height, pack-years, smoking status, systolic blood pressure, use of antihypertensive medications, and prevalent heart failure, myocardial infarction, or stroke at baseline.

**Model 3:** Model 2 with additional adjustment for heart failure, myocardial infarction, or stroke as time-varying covariates.

*Total WBC Count SD = 1.70 x 10 ^**9**^ **/L**

### WBC Differential Count and AF Risk

**Fig 3:**
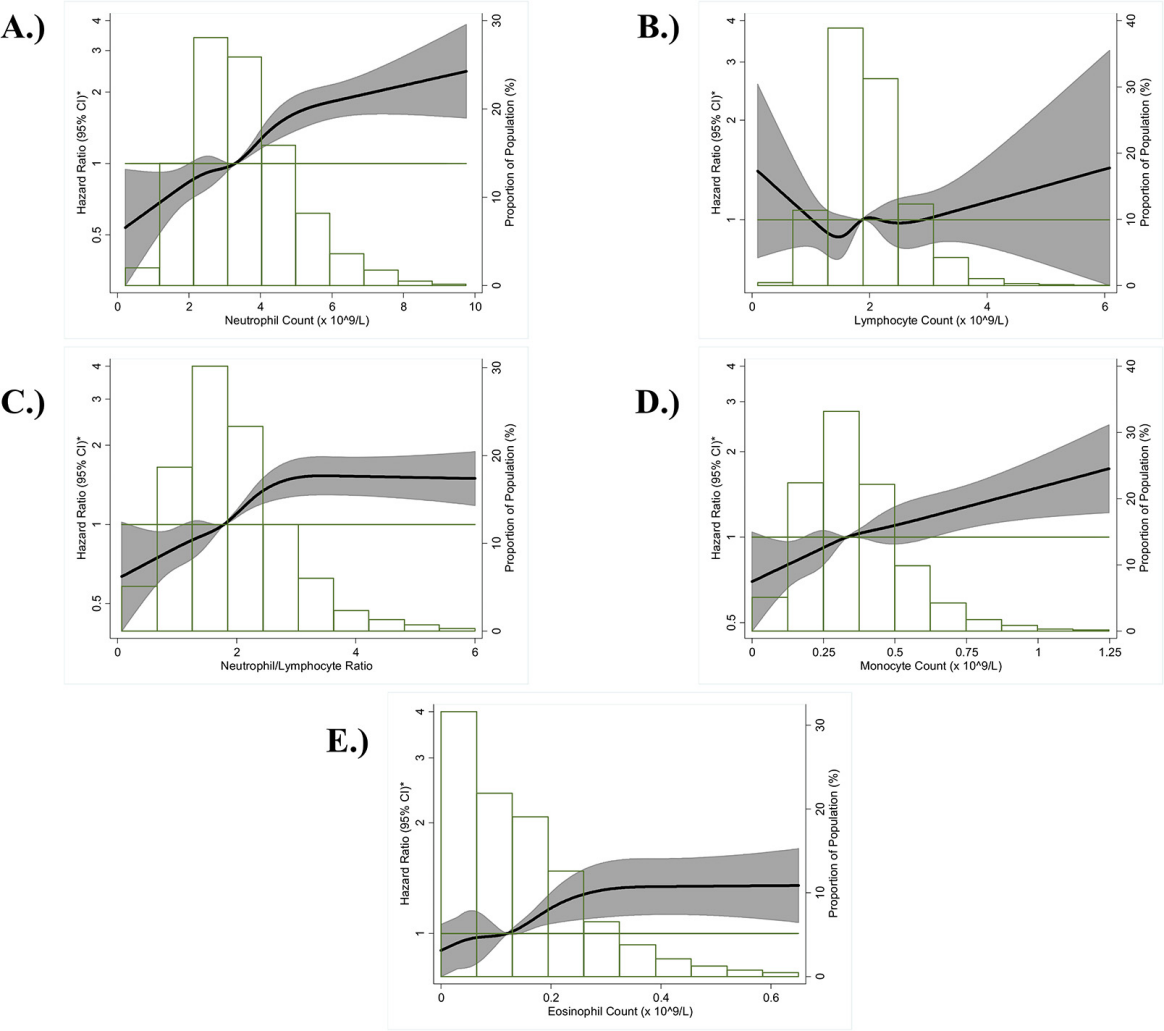
Association between each white blood cell differential count and incident atrial fibrillation presented as hazard ratio (solid line) and 95% confidence intervals (shaded area), Atherosclerosis Risk in Communities Study, 1987 to 2010. (A) Neutrophil Count; (B) Lymphocyte Count; (C) Neutrophil Count/Lymphocyte Count Ratio; (D) Monocyte Count; (E) Eosinophil Count. *Cox proportional hazards model using restricted cubic splines with knots at the 5^th^, 27.5^th^, 50^th^, 72.5^th^ and 95^th^ percentiles and adjustment for age, race, and sex. The reference is the median value of each white blood cell differential count (hazard ratio = 1), and the histogram represents the frequency distribution of each white blood cell differential count in the study sample.

Table 3 shows the associations of each components of WBC differential count (as quintiles/groups and as a 1-SD linear variable) with incident AF. Neutrophil count was positively and linearly associated with AF risk after adjustment for age, race, and sex (Fig 3A). Comparing those in the highest quintile (4.56–9.76 x 10^9^/L) to those in the lowest quintile (0.22–2.30 x 10^9^/L), the adjusted HR for AF potential confounders and a history of CVD was 1.57 (95% CI: 1.28–1.93) (Table 3, model 2). In contrast, lymphocyte count showed a weak, inverse association with AF risk (Fig 3B). After adjustment (Table 3, model 2), the inverse association was strengthened, and those in the highest quintile (>2.44–6.08 x 10^9^/L) had a 30% reduced risk of AF than those in the lowest quintile (0.09–1.44 x 10^9^/L) (HR: 0.70, 95% CI: 0.58–0.84). The neutrophil count/lymphocyte count ratio was positively associated with AF risk (Fig 3C). In Table 3 (model 2), those in the highest neutrophil/lymphocyte ratio quintile (2.57–53.0) had a HR of 1.70 (95% CI: 1.39–2.08) when compared to those in the lowest quintile (0.07–1.18) after adjustment for AF risk factors and a history of CVD. In the fully adjusted model for neutrophil count, lymphocyte count, and neutrophil/lymphocyte ratio (Table 3, model 3), the adjusted HRs for both the quintiles and linear results remained significant though slightly attenuated.

**Table 3:** Hazard ratio (HR) and 95% confidence interval (CI) of atrial fibrillation (AF) by white blood cell differential count, Atherosclerosis Risk in Communities Study, 1987 to 2010.

	**Neutrophil Count (x 10** ^**9**^ **/L)**					
	**0.22–2.30**	**>2.30–2.96**	**2.97–3.60**	**3.61–4.55**	**4.56–9.76**	**Linear**
	**(n = 2141)**	**(n = 2140)**	**(n = 2116)**	**(n = 2132)**	**(n = 2132)**	(per 1-SD* Increase)
**AF Cases**	186	224	249	291	364	1314
**Model 1 HR**	1	1.19	1.34	1.62	2.27	1.32
**(95% CI)**	(Reference)	(0.97–1.45)	(1.09–1.63)	(1.33–1.97)	(1.87–2.75)	(1.25–1.39)
**Model 2 HR**	1	1.12	1.17	1.28	1.57	1.16
**(95% CI)**	(Reference)	(0.92–1.38)	(0.95–1.44)	(1.05–1.57)	(1.28–1.93)	(1.09–1.23)
**Model 3 HR**	1	1.12	1.09	1.20	1.36	1.09
**(95% CI)**	(Reference)	(0.91–1.37)	(0.89–1.34)	(0.98–1.47)	(1.11–1.67)	(1.02–1.15)
	**Lymphocyte Count (x 10** ^**9**^ **/L)**					
	**0.09–1.44**	**>1.44–1.74**	**>1.74–2.03**	**>2.03–2.44**	**>2.44–6.08**	**Linear**
	**(n = 2149)**	**(n = 2123)**	**(n = 2141)**	**(n = 2120)**	**(n = 2128)**	(per 1-SD* Increase)
**AF Cases**	282	252	271	279	230	1314
**Model 1 HR**	1	0.91	0.99	1.10	0.99	1.03
**(95% CI)**	(Reference)	(0.77–1.08)	(0.84–1.18)	(0.93–1.30)	(0.83–1.18)	(0.98–1.09)
**Model 2 HR**	1	0.87	0.88	0.90	0.70	0.91
**(95% CI)**	(Reference)	(0.74–1.04)	(0.75–1.05)	(0.76–1.06)	(0.58–0.84)	(0.86–0.97)
**Model 3 HR**	1	0.86	0.86	0.83	0.67	0.90
**(95% CI)**	(Reference)	(0.72–1.02)	(0.72–1.01)	(0.70–0.99)	(0.56–0.81)	(0.84–0.95)
	**Neutrophil Count/Lymphocyte Count Ratio**					
	**0.07–1.18**	**>1.18–1.58**	**1.59–2.02**	**2.03–2.56**	**2.57–53.0**	**Linear**
	**(n = 2140)**	**(n = 2124)**	**(n = 2238)**	**(n = 2044)**	**(n = 2115)**	(per 1-SD* Increase)
**AF Cases**	184	231	253	306	340	1314
**Model 1 HR**	1	1.24	1.24	1.70	1.92	1.08
**(95% CI)**	(Reference)	(1.02–1.53)	(1.01–1.53)	(1.39–2.08)	(1.58–2.35)	(1.05–1.11)
**Model 2 HR**	1	1.21	1.22	1.58	1.70	1.07
**(95% CI)**	(Reference)	(0.99–1.48)	(0.99–1.50)	(1.29–1.93)	(1.39–2.08)	(1.04–1.10)
**Model 3 HR**	1	1.23	1.22	1.46	1.63	1.07
**(95% CI)**	(Reference)	(1.00–1.51)	(0.99–1.51)	(1.19–1.80)	(1.33–2.00)	(1.04–1.11)
	**Monocyte Count (x 10** ^**9**^ **/L)**					
	**0–0.22**	**>0.22–0.29**	**0.30–0.37**	**>0.37–0.48**	**>0.48–3.72**	**Linear**
	**(n = 2149)**	**(n = 2116)**	**(n = 2145)**	**(n = 2179)**	**(n = 2072)**	(per 1-SD* Increase)
**AF Cases**	212	242	283	279	298	1314
**Model 1 HR**	1	1.11	1.29	1.22	1.47	1.14
**(95% CI)**	(Reference)	(0.93–1.34)	(1.08–1.54)	(1.02–1.46)	(1.23–1.75)	(1.08–1.19)
**Model 2 HR**	1	1.13	1.25	1.12	1.16	1.05
**(95% CI)**	(Reference)	(0.94–1.36)	(1.04–1.49)	(0.93–1.34)	(0.97–1.40)	(1.00–1.11)
**Model 3 HR**	1	1.20	1.28	1.07	1.10	1.02
**(95% CI)**	(Reference)	(0.99–1.44)	(1.07–1.53)	(0.90–1.29)	(0.92–1.32)	(0.97–1.08)
	**Eosinophil Count (x 10** ^**9**^ **/L)**					
	**0**	**0.01–0.09**	**>0.09–0.15**	**>0.15–0.23**	**>0.23–3.16**	**Linear**
	**(n = 2287)**	**(n = 1983)**	**(n = 2137)**	**(n = 2135)**	**(n = 2119)**	(per 1-SD* Increase)
**AF Cases**	257	219	245	284	309	1314
**Model 1 HR**	1	1.06	1.02	1.25	1.38	1.09
**(95% CI)**	(Reference)	(0.87–1.29)	(0.84–1.24)	(1.03–1.52)	(1.14–1.67)	(1.04–1.13)
**Model 2 HR**	1	1.13	1.04	1.16	1.22	1.05
**(95% CI)**	(Reference)	(0.93–1.38)	(0.85–1.27)	(0.95–1.40)	(1.01–1.48)	(1.00–1.10)
**Model 3 HR**	1	1.18	1.06	1.17	1.18	1.03
**(95% CI)**	(Reference)	(0.97–1.44)	(0.87–1.30)	(0.97–1.43)	(0.98–1.44)	(0.98–1.09)
	**Basophil Count (x 10** ^**9**^ **/L)**					
	**0**	**0.01–0.05**	**>0.05–0.62**			**Linear**
	**(n = 5450)**	**(n = 1763)**	**(n = 3448)**			(per 1-SD* Increase)
**AF Cases**	670	169	475			1314
**Model 1 HR**	1	0.72	1.09			1.04
**(95% CI)**	(Reference)	(0.60–0.85)	(0.97–1.23)			(0.98–1.10)
**Model 2 HR**	1	0.87	0.99			0.99
**(95% CI)**	(Reference)	(0.73–1.04)	(0.88–1.12)			(0.94–1.05)
**Model 3 HR**	1	0.94	0.97			0.99
**(95% CI)**	(Reference)	(0.79–1.13)	(0.86–1.10)			(0.94–1.04)

**Model 1:** Cox proportional hazards model adjusted for age, race, sex, and study site.

**Model 2:** Model 1 with additional adjustment for body mass index, chronic obstructive pulmonary disease, diabetes mellitus, drinking status, educational level, height, pack-years, smoking status, systolic blood pressure, use of antihypertensive medications, and prevalent heart failure, myocardial infarction, or stroke at baseline.

**Model 3:** Model 2 with additional adjustment for heart failure, myocardial infarction, or stroke as time-varying covariates.

*Neutrophil Count SD = 1.42 x 10 ^**9**^ **/L, Lymphocyte Count SD = 0.64 x 10** ^**9**^ **/L, Neutrophil/Lymphocyte Ratio SD = 1.25; Monocyte Count SD = 0.18 x 10** ^**9**^ **/L, Eosinophil Count SD = 0.15 x 10** ^**9**^ **/L, Basophil Count SD = 0.04 x 10** ^**9**^ **/L**

Both monocytes and eosinophils had positive associations with incident AF after adjustment for demographics (Fig 3D and 3E). With further adjustment for AF risk factors including CVD history (Table 3, model 2), eosinophils had a positive association with incident AF where the HR of AF for the highest quintile compared to the lower quintile was 1.22 (95% CI: 1.01–1.48). The monocyte-AF association comparing the highest to lowest quintile was attenuated to non-significance. No association was observed between basophil count and AF risk after adjustment for AF risk factors and potential confounders including history of CVD (Table 3, model 2).

### Sensitivity Analyses

We conducted five additional sensitivity analyses. First, those with incident AF events within the first two years of follow-up from baseline were excluded (n = 52). Overall, the HRs for both total WBC count and WBC differential count with incident AF were of a similar magnitude to those seen in the models including all of the incident AF cases in both the quintile and linear results (results not shown). Secondly, fibrinogen and albumin were added to a multivariate model including multiple AF potential confounders and a history of CVD. Additional adjustment for fibrinogen and albumin at baseline further attenuated the associations between total WBC count and AF risk (HR of AF for the highest to lowest quintile: 1.15; 95% CI: 0.98–1.35; HR per 1-SD linear change: 1.06; 95% CI: 1.01–1.12; Table G in S1 File). High fibrinogen and low albumin were each associated with AF after adjustment for total WBC count (HR of AF for fibrinogen per 1-SD linear change: 1.09; 95% CI: 1.04–1.14; HR of albumin per 1-SD linear change: 0.89; 95% CI: 0.85–0.94). However, the HRs for each of the WBC differential count-AF associations with adjustment for fibrinogen and albumin were of a similar magnitude to the models without fibrinogen and albumin (Table G in S1 File). Third, an additional analysis accounting for the competing risk of death was performed, with the results being comparable to the main results (Table H in S1 File). Fourth, AF ascertainment results separated by ECG and hospitalization cases followed similar directionality as the main results (Table I in S1 File). Finally, restricting follow-up to the first ten years resulted in HRs for both total WBC count and WBC differential count with incident AF being of a similar magnitude in both the quintile and linear results (results not shown).

## Discussion

In this prospective analysis of the ARIC cohort, a positive linear association between total WBC count and incident AF was identified, with the highest risk of AF in individuals at the higher end of the distribution of total WBC count. This association was greatly attenuated after further adjustment for risk factors of AF but remained significant, which is similar to previous literature examining this association in the general population.[14,15] For WBC differential, elevated neutrophil and monocyte counts were positively associated with AF risk while an inverse association was identified between lymphocyte count and AF after adjustment. A higher neutrophil count/lymphocyte count ratio was also positively associated with AF risk. No association was identified between eosinophils or basophils and AF. These results did not differ significantly by race, sex, or age.

These results from the ARIC Study are comparable to the results from recent studies that were conducted mainly on white populations. The Framingham Heart Study and the Norwegian Tromsø study[14,15] reported a positive association between total WBC count and AF risk. However, in the Framingham Heart Study, the observed positive association became non-significant after extending the follow-up period to ten years although the magnitude of the results were similar to those after 5 years of follow-up.[14] The Tromsø study examined many systematic inflammatory biomarkers and identified a positive association between total WBC count and incident AF in the full cohort after a mean follow-up of 10 years and adjustment for AF risk factors and potential confounders. This study found no association between any of the WBC differential subtypes and AF, though.[15]

Although both studies reached a similar conclusion in respect to total WBC count and incident AF, the ARIC study was able to address additional issues raised by the data limitations of each study. In the ARIC cohort, the positive association between total WBC count and AF remained present with a longer median follow-up of 21.5 years and adjustment for many AF risk factors and potential confounders. A racial comparison between whites and African Americans was possible in our analysis, and no significant interaction by race was identified. In contrast to the Tromsø study, the ARIC study had information about prevalent or incident HF, an important risk factor for AF, and we found significant associations between WBC differential and AF risk. The larger sample size and statistical power of the ARIC study may have a role in the differences between studies although further investigation is warranted.

WBC differential, specifically neutrophils, may play a pathophysiological role on the mechanical pathway between inflammation and AF. Previous evidence suggests that the enzyme MPO, produced abundantly by neutrophils, may be involved in the development of atrial fibrosis, resulting in an increased risk of AF.[18,19] Atrial fibrosis is a major component of the structural remodeling that constitutes the substrate for AF[31], possibly linking inflammation and AF, and eventually leading to the initiation and propagation of AF.[32–34] Matrix metalloproteinases (MMPs) are enzymes involved in the turnover of extracellular matrix (ECM) proteins within the atria, a key process in the development of atrial fibrosis. MMPs have been associated with ECM remodeling in AF,[35] and circulating levels of some MMPs have been associated with AF risk.[36] MMPs, in turn, have their activity regulated by redox reactions. Neutrophil-secreted MPOs, through generation of hypocholorous acid, regulate MMP activity through these redox reactions,[37,38] and, as a result, they affect the pathway promoting atrial fibrosis and eventually AF. Our results from the ARIC study provide support for this hypothesis. However, more research is necessary to refine our understanding of this pathway. In the ARIC study, we also found an inverse association between lymphocyte counts and AF. To our knowledge, no clear mechanism can explain this association; therefore, further exploration is needed as to determine if this novel finding is due to chance or if it has pathophysiological merits.

A major limitation in this analysis is the method of AF ascertainment. Most AF cases were identified through hospitalization discharges, which omit asymptomatic AF or AF cases managed in outpatient settings. Therefore, under-ascertainment of incident AF may have occurred. Despite this limitation, the ARIC study and other cohorts have shown acceptable validity of discharge code-based AF diagnosis.[27,39] In addition, rates of AF in the ARIC cohort are similar to those from other population-based studies, improving confidence in the validity of the AF case ascertainment.[27]

For the current analysis, we used single baseline measurements of total WBC and differential counts. These counts may possibly have changed during follow-up, which may not reflect the total WBC or differential count level immediately preceding an AF event. The results from the first 10 years of follow-up were similar to those for the entire follow-up period. However, multiple repeated counts over time may be more relevant and accurate as a predictor of AF. Also, the ARIC study does not have information on some inflammatory biomarkers, such as high-sensitivity C-reactive protein, measured at the baseline visit. Results were mostly unaffected after adjustment for available inflammatory biomarkers, such as fibrinogen and albumin. Individuals with elevated neutrophil counts linked to ongoing infections were not excluded due to a lack of information concerning ongoing infections in ARIC at baseline; however, those who may have had serious ongoing infections at baseline would have most likely not included in the ARIC study, or the total number would most likely be very low that it would not explain the association. Any link between elevated neutrophil count due to systemic inflammatory diseases such as rheumatoid arthritis, inflammatory bowel disease, or psoriasis was not examined due to a lack of data on those conditions in ARIC, though the numbers would also be too low to explain the association. No data was available regarding how long neutrophils were elevated at baseline as well. Finally, unmeasured or residual confounding could also partly explain the observed association through other means such as other systemic inflammatory biomarkers or diseases not measured in the ARIC study. Despite these limitations, several strengths should be highlighted, including the large, biracial sample with an extensive follow-up time, a large number of AF events, and comprehensive measurement of key cardiovascular covariates.

## Conclusion

Overall, this study has identified a positive association between high total WBC, neutrophil, and monocyte counts, separately, with higher AF risk and an inverse association between lymphocyte count with AF risk. The positive association between neutrophil count and AF seems to support the hypothesis that MPO may play a role in the development of atrial fibrosis and eventually AF. Systemic inflammation may underlie this association and requires further investigation to examine the specific mechanisms. Future research should also examine the association of inflammatory markers with atrial fibrosis in the general population, and the potential impact of specific anti-inflammatory treatments in reducing AF risk.

## Supporting Information

S1 File WBC-AF supporting information. Tables A-I.
(DOCX)
