# Supplementary material for: Association of White Blood Cell Count and Differential with the Incidence of Atrial Fibrillation: The Atherosclerosis Risk in Communities (ARIC) Study
Source: PLoS One. 2015 Aug 27;10(8):e0136219. doi: 10.1371/journal.pone.0136219 (PMC4551739; doi:10.1371/journal.pone.0136219)
Supplement: S1 File — Tables A-I. (DOCX) [file pone.0136219.s001.docx]

**Table A. Baseline characteristics by neutrophil count quintile, Atherosclerosis Risk in Communities Study, 1987 to 1989**

|  | **Neutrophil Count (x 10^9^/L)†** | | | | |
| --- | --- | --- | --- | --- | --- |
|  | **0.22-2.30** | **>2.30-2.96** | **2.97-3.60** | **3.61-4.55** | **4.56-9.76** |
|  | **(n=2141)** | **(n=2140)** | **(n=2116)** | **(n=2132)** | **(n=2132)** |
| Neutrophil count median, x 10^9^/L | 1.8 | 2.7 | 3.3 | 4.0 | 5.4 |
| Age, years | 53.6 (5.7) | 54.0 (5.7) | 54.0 (5.7) | 54.2 (5.9) | 54.1 (5.9) |
| Females, % | 62.2 | 59.2 | 55.8 | 51.2 | 49.0 |
| African Americans, % | 70.0 | 35.9 | 22.4 | 20.4 | 17.3 |
| Body mass index, kg/m^2^ | 28.1 (5.6) | 27.5 (5.4) | 27.2 (5.1) | 27.8 (5.3) | 27.5 (5.4) |
| Chronic obstructive pulmonary disease, % | 6.3 | 7.7 | 8.9 | 10.1 | 13.0 |
| Current drinker, % | 42.0 | 58.5 | 61.9 | 62.3 | 64.1 |
| Current smoker, % | 17.2 | 17.2 | 19.5 | 28.9 | 50.4 |
| Pack-years | 9.8 (18.0) | 11.2 (17.6) | 13.9 (19.3) | 18.8 (23.0) | 25.7 (24.4) |
| Diabetes, % | 10.4 | 10.3 | 10.0 | 12.6 | 14.7 |
| Height, cm | 168.4 (9.2) | 168.4 (9.1) | 168.9 (9.3) | 169.4 (9.6) | 169.4 (9.3) |
| High school degree, % | 33.3 | 37.4 | 43.4 | 41.1 | 43.3 |
| Systolic BP, mmHg | 123.5 (19.9) | 121.2 (19.4) | 121.1 (18.5) | 121.7 (18.5) | 121.3 (19.5) |
| Use of antihypertensive medications, % | 35.0 | 27.2 | 26.9 | 27.5 | 31.8 |
| Prevalent heart failure, % | 5.0 | 3.4 | 3.7 | 3.8 | 6.5 |
| Prevalent myocardial infarction, % | 2.1 | 2.7 | 3.1 | 4.2 | 5.5 |
| Prevalent stroke, % | 1.7 | 1.4 | 1.9 | 1.7 | 2.3 |
| Basophil count, x 10^9^/L | 0.03 (0.04) | 0.03 (0.04) | 0.03 (0.04) | 0.04 (0.05) | 0.04 (0.05) |
| Eosinophil count, x 10^9^/L | 0.1 (0.1) | 0.1 (0.1) | 0.1 (0.1) | 0.1 (0.2) | 0.2 (0.2) |
| Lymphocyte count, x 10^9^/L | 2.0 (0.7) | 1.8 (0.6) | 1.9 (0.6) | 2.0 (0.6) | 2.1 (0.7) |
| Monocyte count, x 10^9^/L | 0.3 (0.2) | 0.3 (0.1) | 0.3 (0.2) | 0.4 (0.2) | 0.5 (0.2) |
| Neutrophil/lymphocyte ratio | 1.0 (0.5) | 1.6 (0.5) | 1.9 (0.6) | 2.3 (1.1) | 3.0 (1.9) |
| Total WBC count, x 10^9^/L | 4.3 (0.8) | 5.0 (0.8) | 5.7 (0.7) | 6.6 (0.8) | 8.5 (1.3) |

**BP indicates blood pressure. WBC indicates white blood cell. Values are mean (SD) when appropriate.**

**†All baseline characteristics have p-values <0.05 for differences in means (ANOVA) and percentages (Chi-Square) between neutrophil quintiles except for prevalent stroke.**

**Table B. Baseline characteristics by lymphocyte count quintile, Atherosclerosis Risk in Communities Study, 1987 to 1989**

|  | **Lymphocyte Count (x 10^9^/L)†** | | | | |
| --- | --- | --- | --- | --- | --- |
|  | **0.09-1.44** | **>1.44-1.74** | **>1.74-2.03** | **>2.03-2.44** | **>2.44-6.08** |
|  | **(n=2149)** | **(n=2123)** | **(n=2141)** | **(n=2120)** | **(n=2128)** |
| Lymphocyte count median, x 10^9^/L | 1.3 | 1.6 | 1.9 | 2.2 | 2.8 |
| Age, years | 54.1 (5.9) | 54.0 (5.9) | 54.1 (5.9) | 54.0 (5.7) | 53.7 (5.7) |
| Females, % | 52.1 | 53.6 | 54.1 | 57.1 | 60.5 |
| African Americans, % | 23.4 | 25.1 | 30.5 | 36.5 | 51.0 |
| Body mass index, kg/m^2^ | 26.3 (4.7) | 27.0 (4.9) | 27.7 (5.3) | 28.1 (5.4) | 29.1 (5.9) |
| Chronic obstructive pulmonary disease, % | 7.4 | 7.3 | 9.4 | 10.3 | 11.6 |
| Current drinker, % | 63.6 | 62.7 | 56.8 | 55.5 | 50.1 |
| Current smoker, % | 14.0 | 18.6 | 23.4 | 33.4 | 43.9 |
| Pack-years | 11.8 (19.3) | 12.9 (18.6) | 15.9 (21.6) | 18.4 (23.2) | 20.3 (22.9) |
| Diabetes, % | 8.0 | 8.9 | 11.1 | 12.4 | 17.7 |
| Height, cm | 169.6 (9.5) | 169.4 (9.4) | 169.0 (9.3) | 168.5 (9.3) | 167.9 (9.1) |
| High school degree, % | 40.9 | 39.9 | 39.7 | 39.9 | 38.1 |
| Systolic BP, mmHg | 120.5 (18.9) | 120.7 (18.9) | 121.9 (19.2) | 121.7 (18.8) | 124.2 (19.8) |
| Use of antihypertensive medications, % | 27.0 | 27.0 | 28.1 | 29.5 | 36.8 |
| Prevalent heart failure, % | 3.1 | 3.2 | 4.4 | 5.0 | 6.8 |
| Prevalent myocardial infarction, % | 3.2 | 2.2 | 3.3 | 4.0 | 4.8 |
| Prevalent stroke, % | 1.8 | 1.9 | 1.3 | 1.7 | 2.4 |
| Basophil count, x 10^9^/L | 0.03 (0.03) | 0.03 (0.04) | 0.03 (0.04) | 0.04 (0.04) | 0.04 (0.05) |
| Eosinophil count, x 10^9^/L | 0.1 (0.1) | 0.1 (0.1) | 0.1 (0.1) | 0.2 (0.2) | 0.2 (0.2) |
| Monocyte count, x 10^9^/L | 0.3 (0.1) | 0.3 (0.2) | 0.4 (0.2) | 0.4 (0.2) | 0.4 (0.2) |
| Neutrophil count, x 10^9^/L | 3.3 (1.3) | 3.3 (1.3) | 3.5 (1.4) | 3.6 (1.5) | 3.7 (1.6) |
| Neutrophil/lymphocyte ratio | 2.9 (2.1) | 2.1 (0.8) | 1.9 (0.7) | 1.6 (0.7) | 1.3 (0.6) |
| Total WBC count, x 10^9^/L | 5.0 (1.3) | 5.5 (1.3) | 6.0 (1.5) | 6.4 (1.5) | 7.4 (1.8) |

**BP indicates blood pressure. WBC indicates white blood cell. Values are mean (SD) when appropriate.**

**†All baseline characteristics have p-values <0.05 for differences in means (ANOVA) and percentages (Chi-Square) between lymphocyte quintiles except for age and prevalent stroke.**

**Table C. Baseline characteristics by neutrophil count/lymphocyte count ratio quintile, Atherosclerosis Risk in Communities Study, 1987 to 1989**

|  | **Neutrophil Count/Lymphocyte Count Ratio†** | | | | |
| --- | --- | --- | --- | --- | --- |
|  | **0.07-1.77** | **1.78-1.58** | **1.59-2.02** | **2.03-2.56** | **2.57-53.0** |
|  | **(n=2140)** | **(n=2124)** | **(n=2238)** | **(n=2044)** | **(n=2115)** |
| Neutrophil/lymphocyte ratio median | 0.9 | 1.4 | 1.8 | 2.3 | 3.1 |
| Age, years | 53.5 (5.6) | 54.1 (5.8) | 54.1 (5.7) | 54.0 (5.9) | 54.2 (6.0) |
| Females, % | 64.8 | 59.8 | 53.4 | 50.8 | 48.5 |
| African Americans, % | 74.9 | 37.1 | 22.5 | 17.2 | 14.1 |
| Body mass index, kg/m^2^ | 29.0 (5.9) | 27.8 (5.4) | 27.3 (5.0) | 27.1 (5.2) | 26.8 (5.0) |
| Chronic obstructive pulmonary disease, % | 7.2 | 9.5 | 10.1 | 8.8 | 10.4 |
| Current drinker, % | 40.4 | 56.4 | 61.6 | 63.9 | 66.6 |
| Current smoker, % | 24.4 | 24.3 | 24.8 | 26.7 | 33.1 |
| Pack-years | 12.0 (19.2) | 14.6 (20.4) | 16.0 (21.5) | 16.6 (21.7) | 20.0 (23.3) |
| Diabetes, % | 14.3 | 11.4 | 10.0 | 10.8 | 11.5 |
| Height, cm | 167.8 (9.0) | 168.4 (9.3) | 169.1 (9.4) | 169.5 (9.6) | 169.7 (9.3) |
| High school degree, % | 32.7 | 39.7 | 40.8 | 41.9 | 43.4 |
| Systolic BP, mmHg | 124.6 (20.1) | 122.0 (18.8) | 120.4 (18.8) | 120.5 (18.1) | 121.4 (19.6) |
| Use of antihypertensive medications, % | 37.4 | 29.9 | 25.7 | 26.4 | 29.2 |
| Prevalent heart failure, % | 5.9 | 4.4 | 3.6 | 3.6 | 4.9 |
| Prevalent myocardial infarction, % | 2.9 | 3.1 | 3.2 | 3.6 | 4.7 |
| Prevalent stroke, % | 1.7 | 1.7 | 1.8 | 2.1 | 1.7 |
| Basophil count, x 10^9^/L | 0.03 (0.04) | 0.04 (0.04) | 0.04 (0.04) | 0.03 (0.04) | 0.03 (0.05) |
| Eosinophil count, x 10^9^/L | 0.1 (0.1) | 0.1 (0.2) | 0.2 (0.1) | 0.1 (0.1) | 0.1 (0.1) |
| Lymphocyte count, x 10^9^/L | 2.5 (0.7) | 2.1 (0.6) | 1.9 (0.5) | 1.8 (0.5) | 1.5 (0.4) |
| Monocyte count, x 10^9^/L | 0.3 (0.2) | 0.3 (0.2) | 0.4 (0.2) | 0.4 (0.2) | 0.4 (0.2) |
| Neutrophil count, x 10^9^/L | 2.0 (0.7) | 3.0 (0.8) | 3.5 (0.9) | 4.0 (1.1) | 5.0 (1.4) |
| Total WBC count, x 10^9^/L | 5.1 (1.4) | 5.7 (1.5) | 6.0 (1.6) | 6.4 (1.7) | 7.1 (1.8) |

**BP indicates blood pressure. WBC indicates white blood cell. Values are mean (SD) when appropriate.**

**†All baseline characteristics have p-values <0.05 for differences in means (ANOVA) and percentages (Chi-Square) between neutrophil count/lymphocyte count ratio quintiles except for basophil count, eosinophil count, and prevalent stroke.**

**Table D. Baseline characteristics by monocyte count quintile, Atherosclerosis Risk in Communities Study, 1987 to 1989**

|  | **Monocyte Count (x 10^9^/L)†** | | | | |
| --- | --- | --- | --- | --- | --- |
|  | **0-0.22** | **>0.22-0.29** | **0.30-0.37** | **>0.37-0.48** | **>0.48-3.72** |
|  | **(n=2149)** | **(n=2116)** | **(n=2145)** | **(n=2179)** | **(n=2072)** |
| Monocyte count median, x 10^9^/L | 0.2 | 0.3 | 0.3 | 0.4 | 0.6 |
| Age, years | 53.7 (5.7) | 53.8 (5.8) | 54.0 (5.7) | 54.2 (5.9) | 54.3 (5.8) |
| Females, % | 64.3 | 62.0 | 56.5 | 49.2 | 45.2 |
| African Americans, % | 39.8 | 37.4 | 31.8 | 28.3 | 29.0 |
| Body mass index, kg/m^2^ | 27.6 (5.2) | 27.5 (5.4) | 27.6 (5.4) | 27.7 (5.5) | 27.8 (5.3) |
| Chronic obstructive pulmonary disease, % | 6.8 | 8.6 | 9.7 | 10.1 | 10.8 |
| Current drinker, % | 55.8 | 54.3 | 57.2 | 59.2 | 62.3 |
| Current smoker, % | 19.9 | 20.1 | 23.6 | 29.2 | 40.6 |
| Pack-years | 11.7 (19.2) | 11.9 (18.2) | 14.5 (20.5) | 18.6 (22.6) | 22.7 (24.1) |
| Diabetes, % | 10.5 | 11.6 | 10.1 | 11.6 | 14.2 |
| Height, cm | 167.8 (9.2) | 168.1 (9.0) | 168.8 (9.4) | 169.8 (9.5) | 170.0 (9.4) |
| High school degree, % | 40.5 | 40.3 | 38.8 | 39.5 | 39.3 |
| Systolic BP, mmHg | 121.1 (19.2) | 121.4 (18.9) | 122.0 (19.5) | 122.0 (18.9) | 122.5 (19.3) |
| Use of antihypertensive medications, % | 29.7 | 29.8 | 27.2 | 28.8 | 33.1 |
| Prevalent heart failure, % | 3.7 | 4.2 | 3.8 | 4.6 | 6.1 |
| Prevalent myocardial infarction, % | 2.6 | 2.2 | 3.2 | 4.1 | 5.5 |
| Prevalent stroke, % | 2.3 | 1.3 | 1.8 | 1.7 | 1.9 |
| Basophil count, x 10^9^/L | 0.03 (0.04) | 0.04 (0.04) | 0.03 (0.04) | 0.03 (0.04) | 0.03 (0.05) |
| Eosinophil count, x 10^9^/L | 0.1 (0.1) | 0.1 (0.1) | 0.1 (0.1) | 0.2 (0.2) | 0.2 (0.2) |
| Lymphocyte count, x 10^9^/L | 1.9 (0.6) | 1.8 (0.6) | 1.9 (0.6) | 2.0 (0.6) | 2.2 (0.7) |
| Neutrophil count, x 10^9^/L | 2.9 (1.2) | 3.0 (1.1) | 3.4 (1.2) | 3.8 (1.4) | 4.3 (1.6) |
| Neutrophil/Lymphocyte ratio | 1.8 (1.5) | 1.8 (0.9) | 2.0 (1.4) | 2.0 (1.0) | 2.2 (1.2) |
| Total WBC count, x 10^9^/L | 5.1 (1.4) | 5.4 (1.3) | 5.9 (1.4) | 6.4 (1.6) | 7.4 (1.8) |

**BP indicates blood pressure. WBC indicates white blood cell. Values are mean (SD) when appropriate.**

**†All baseline characteristics have p-values <0.05 for differences in means (ANOVA) and percentages (Chi-Square) between monocyte quintiles except for body mass index, education level, systolic BP, prevalent stroke, and basophil count.**

**Table E. Baseline characteristics by eosinophil count quintile, Atherosclerosis Risk in Communities Study, 1987 to 1989**

|  | **Eosinophil Count (x 10^9^/L)†** | | | | |
| --- | --- | --- | --- | --- | --- |
|  | **0** | **0.01-0.09** | **>0.09-0.15** | **>0.15-0.23** | **>0.23-3.16** |
|  | **(n=2287)** | **(n=1983)** | **(n=2137)** | **(n=2135)** | **(n=2119)** |
| Eosinophil count median, x 10^9^/L | 0 | 0.1 | 0.1 | 0.2 | 0.3 |
| Age, years | 54.0 (5.6) | 53.6 (5.7) | 54.2 (5.9) | 53.9 (5.8) | 54.2 (5.9) |
| Females, % | 52.7 | 62.4 | 58.9 | 54.5 | 49.7 |
| African Americans, % | 13.4 | 45.1 | 40.2 | 34.6 | 35.2 |
| Body mass index, kg/m^2^ | 27.2 (4.8) | 27.4 (5.3) | 27.7 (5.4) | 27.9 (5.6) | 27.9 (5.7) |
| Chronic obstructive pulmonary disease, % | 9.1 | 6.3 | 8.4 | 9.9 | 12.0 |
| Current drinker, % | 75.2 | 51.6 | 51.8 | 53.3 | 55.1 |
| Current smoker, % | 21.3 | 23.1 | 24.2 | 30.2 | 34.6 |
| Pack-years | 15.6 (20.7) | 13.2 (20.0) | 13.8 (20.3) | 17.2 (22.9) | 19.3 (22.5) |
| Diabetes, % | 8.9 | 11.4 | 11.4 | 12.6 | 13.9 |
| Height, cm | 169.2 (9.4) | 167.8 (9.0) | 168.4 (9.1) | 169.1 (9.7) | 169.8 (9.4) |
| High school degree, % | 46.0 | 38.7 | 38.1 | 37.9 | 37.1 |
| Systolic BP, mmHg | 120.3 (17.4) | 122.6 (20.4) | 121.8 (18.9) | 122.3 (20.1) | 122.1 (19.0) |
| Use of antihypertensive medications, % | 24.2 | 29.6 | 30.1 | 32.2 | 32.9 |
| Prevalent heart failure, % | 3.7 | 3.5 | 4.3 | 4.9 | 6.0 |
| Prevalent myocardial infarction, % | 3.5 | 2.7 | 2.9 | 4.0 | 4.5 |
| Prevalent stroke, % | 1.1 | 1.7 | 2.0 | 2.2 | 2.1 |
| Basophil count, x 10^9^/L | 0.01 (0.03) | 0.03 (0.04) | 0.04 (0.04) | 0.04 (0.04) | 0.05 (0.05) |
| Lymphocyte count, x 10^9^/L | 1.9 (0.6) | 1.9 (0.6) | 1.9 (0.6) | 2.0 (0.6) | 2.1 (0.7) |
| Monocyte count, x 10^9^/L | 0.4 (0.2) | 0.3 (0.2) | 0.3 (0.1) | 0.4 (0.2) | 0.4 (0.2) |
| Neutrophil count, x 10^9^/L | 3.7 (1.4) | 3.1 (1.3) | 3.2 (1.3) | 3.5 (1.4) | 3.8 (1.5) |
| Neutrophil/Lymphocyte ratio | 2.2 (1.7) | 1.9 (1.4) | 1.8 (0.9) | 1.9 (1.0) | 2.0 (1.0) |
| Total WBC count, x 10^9^/L | 6.1 (1.7) | 5.5 (1.5) | 5.6 (1.5) | 6.2 (1.7) | 6.8 (1.8) |

**BP indicates blood pressure. WBC indicates white blood cell. Values are mean (SD) when appropriate.**

**†All baseline characteristics have p-values <0.05 for differences in means (ANOVA) and percentages (Chi-Square) between eosinophil quintiles.**

**Table F. Baseline characteristics by basophil count group, Atherosclerosis Risk in Communities Study, 1987 to 1989**

|  | **Basophil Count (x 10^9^/L)†** | | |
| --- | --- | --- | --- |
|  | **0** | **0.01-0.05** | **>0.05-0.62** |
|  | **(n=5450)** | **(n=1763)** | **(n=3448)** |
| Basophil count median, x 10^9^/L | 0 | 0.05 | 0.07 |
| Age, years | 53.9 (5.8) | 53.9 (5.8) | 54.1 (5.9) |
| Females, % | 54.6 | 58.1 | 55.5 |
| African Americans, % | 28.5 | 48.4 | 33.0 |
| Body mass index, kg/m^2^ | 27.6 (5.3) | 27.2 (5.3) | 27.8 (5.5) |
| Chronic obstructive pulmonary disease, % | 9.0 | 7.5 | 10.3 |
| Current drinker, % | 62.0 | 49.2 | 55.3 |
| Current smoker, % | 25.9 | 15.1 | 33.7 |
| Pack-years | 16.1 (21.5) | 10.0 (17.2) | 18.5 (22.7) |
| Diabetes, % | 11.0 | 9.1 | 13.8 |
| Height, cm | 168.9 (9.4) | 169.0 (9.3) | 168.9 (9.3) |
| High school degree, % | 41.3 | 36.6 | 38.7 |
| Systolic BP, mmHg | 121.5 (18.6) | 121.3 (19.5) | 122.4 (19.9) |
| Use of antihypertensive medications, % | 28.1 | 29.0 | 32.6 |
| Prevalent heart failure, % | 4.3 | 3.2 | 5.5 |
| Prevalent myocardial infarction, % | 3.5 | 1.9 | 4.4 |
| Prevalent stroke, % | 1.7 | 1.8 | 2.1 |
| Eosinophil count, x 10^9^/L | 0.1 (0.2) | 0.1 (0.1) | 0.2 (0.1) |
| Lymphocyte count, x 10^9^/L | 2.0 (0.7) | 1.6 (0.4) | 2.1 (0.6) |
| Monocyte count, x 10^9^/L | 0.4 (0.2) | 0.3 (0.1) | 0.4 (0.2) |
| Neutrophil count, x 10^9^/L | 3.6 (1.5) | 2.3 (0.6) | 3.9 (1.3) |
| Neutrophil/Lymphocyte ratio | 2.0 (1.5) | 1.6 (0.7) | 2.0 (1.1) |
| Total WBC count, x 10^9^/L | 6.1 (1.8) | 4.5 (0.6) | 6.7 (1.5) |

**BP indicates blood pressure. WBC indicates white blood cell. Values are mean (SD) when appropriate.**

**†All baseline characteristics have p-values <0.05 for differences in means (ANOVA) and percentages (Chi-Square) between basophil groups except for age, height, systolic BP, and prevalent stroke.**

**Table G. Hazard ratio (HR) and 95% confidence interval (CI) of atrial fibrillation (AF) by total white blood cell (WBC) count and WBC differential count adjusting for other inflammatory markers (fibrinogen and albumin), Atherosclerosis Risk in Communities Study, 1987 to 2010**

|  | **Total WBC Count (x 10^9^/L)** | | | | | |
| --- | --- | --- | --- | --- | --- | --- |
|  | **3.0-4.6** | **4.7-5.4** | **5.5-6.2** | **6.3-7.4** | **7.5-12.0** | **Linear** |
|  | **(n=2945)** | **(n=2947)** | **(n=2749)** | **(n=2982)** | **(n=2778)** | **(per 1-SD* Increase)** |
| **AF Cases** | 299 | 326 | 373 | 452 | 470 | 1920 |
| **Model HR** | 1 | 0.99 | 1.06 | 1.13 | 1.15 | 1.06 |
| **(95% CI)** | (Reference) | (0.84-1.15) | (0.91-1.24) | (0.97-1.32) | (0.98-1.35) | (1.01-1.12) |
|  | **Neutrophil Count (x 10^9^/L)** | | | | | |
|  | **0.22-2.30** | **>2.30-2.96** | **2.97-3.60** | **3.61-4.55** | **4.56-9.76** | **Linear** |
|  | **(n=2108)** | **(n=2114)** | **(n=2104)** | **(n=2122)** | **(n=2118)** | **(per 1-SD* Increase)** |
| **AF Cases** | 184 | 223 | 249 | 290 | 361 | 1307 |
| **Model HR** | 1 | 1.13 | 1.16 | 1.23 | 1.50 | 1.13 |
| **(95% CI)** | (Reference) | (0.92-1.38) | (0.94-1.42) | (1.00-1.51) | (1.21-1.84) | (1.06-1.20) |
|  | **Lymphocyte Count (x 10^9^/L)** | | | | | |
|  | **0.09-1.44** | **>1.44-1.74** | **>1.74-2.03** | **>2.03-2.44** | **>2.44-6.08** | **Linear** |
|  | **(n=2138)** | **(n=2112)** | **(n=2121)** | **(n=2099)** | **(n=2096)** | **(per 1-SD* Increase)** |
| **AF Cases** | 282 | 249 | 270 | 277 | 229 | 1307 |
| **Model HR** | 1 | 0.86 | 0.89 | 0.89 | 0.69 | 0.91 |
| **(95% CI)** | (Reference) | (0.73-1.02) | (0.75-1.06) | (0.75-1.05) | (0.58-0.83) | (0.86-0.97) |
|  | **Neutrophil Count/Lymphocyte Count Ratio** | | | | | |
|  | **0.07-1.18** | **>1.18-1.58** | **1.59-2.02** | **2.03-2.56** | **2.57-53.0** | **Linear** |
|  | **(n=2099)** | **(n=2105)** | **(n=2226)** | **(n=2033)** | **(n=2103)** | **(per 1-SD* Increase)** |
| **AF Cases** | 183 | 229 | 253 | 305 | 337 | 1307 |
| **Model HR** | 1 | 1.18 | 1.19 | 1.53 | 1.61 | 1.07 |
| **(95% CI)** | (Reference) | (0.96-1.45) | (0.97-1.47) | (1.25-1.88) | (1.31-1.98) | (1.03-1.10) |
|  | **Monocyte Count (x 10^9^/L)** | | | | | |
|  | **0-0.22** | **>0.22-0.29** | **0.30-0.37** | **>0.37-0.48** | **>0.48-3.72** | **Linear** |
|  | **(n=2128)** | **(n=2098)** | **(n=2126)** | **(n=2162)** | **(n=2052)** | **(per 1-SD* Increase)** |
| **AF Cases** | 212 | 241 | 280 | 279 | 295 | 1307 |
| **Model HR** | 1 | 1.12 | 1.23 | 1.09 | 1.10 | 1.04 |
| **(95% CI)** | (Reference) | (0.93-1.36) | (1.03-1.47) | (0.91-1.30) | (0.92-1.32) | (0.98-1.09) |
|  | **Eosinophil Count (x 10^9^/L)** | | | | | |
|  | **0** | **0.01-0.09** | **>0.09-0.15** | **>0.15-0.23** | **>0.23-3.16** | **Linear** |
|  | **(n=2279)** | **(n=1967)** | **(n=2115)** | **(n=2116)** | **(n=2089)** | **(per 1-SD* Increase)** |
| **AF Cases** | 257 | 219 | 245 | 282 | 304 | 1307 |
| **Model HR** | 1 | 1.14 | 1.05 | 1.12 | 1.17 | 1.03 |
| **(95% CI)** | (Reference) | (0.93-1.39) | (0.86-1.28) | (0.92-1.36) | (0.96-1.41) | (0.98-1.09) |
|  | **Basophil Count (x 10^9^/L)** | | | | | |
|  | **0** | | **0.01-0.05** | **>0.05-0.62** | | **Linear** |
|  | **(n=5398)** | | **(n=1744)** | **(n=3424)** | | **(per 1-SD* Increase)** |
| **AF Cases** | 667 | | 168 | 472 | | 1307 |
| **Model HR** | 1 | | 0.88 | 0.98 | | 0.99 |
| **(95% CI)** | (Reference) | | (0.74-1.05) | (0.87-1.11) | | (0.93-1.04) |

**Model:** Cox proportional hazards model adjusted for age, race, sex, study site, body mass index, chronic obstructive pulmonary disease, diabetes mellitus, drinking status, educational level, height, pack-years, smoking status, systolic blood pressure, use of antihypertensive medications, prevalent heart failure, myocardial infarction, or stroke at baseline, fibrinogen, and albumin.

***Total WBC Count SD=1.70 x 10^9^/L, Neutrophil Count SD=1.42 x 10^9^/L, Lymphocyte Count SD=0.64 x 10^9^/L, Neutrophil/Lymphocyte Ratio SD=1.25, Monocyte Count SD=0.18 x 10^9^/L, Eosinophil Count SD=0.15 x 10^9^/L, Basophil Count SD=0.04 x 10^9^/L**

**Table H. Hazard ratio (HR) and 95% confidence interval (CI) of atrial fibrillation (AF) by total white blood cell (WBC) count and WBC differential count adjusting for the competing risk of death, Atherosclerosis Risk in Communities Study, 1987 to 2010**

|  | **Total WBC Count (x 10^9^/L)** | | | | | |
| --- | --- | --- | --- | --- | --- | --- |
|  | **3.0-4.6** | **4.7-5.4** | **5.5-6.2** | **6.3-7.4** | **7.5-12.0** | **Linear** |
|  | **(n=2970)** | **(n=2966)** | **(n=2769)** | **(n=3002)** | **(n=2793)** | **(per 1-SD* Increase)** |
| **AF Cases** | 300 | 326 | 377 | 453 | 472 | 1928 |
| **Model HR** | 1 | 0.97 | 1.09 | 1.10 | 1.14 | 1.05 |
| **(95% CI)** | (Reference) | (0.83-1.14) | (0.93-1.27) | (0.95-1.29) | (0.97-1.34) | (1.00-1.11) |
|  | **Neutrophil Count (x 10^9^/L)** | | | | | |
|  | **0.22-2.30** | **>2.30-2.96** | **2.97-3.60** | **3.61-4.55** | **4.56-9.76** | **Linear** |
|  | **(n=2141)** | **(n=2140)** | **(n=2116)** | **(n=2132)** | **(n=2132)** | **(per 1-SD* Increase)** |
| **AF Cases** | 186 | 224 | 249 | 291 | 364 | 1314 |
| **Model HR** | 1 | 1.09 | 1.12 | 1.19 | 1.40 | 1.10 |
| **(95% CI)** | (Reference) | (0.89-1.33) | (0.92-1.37) | (0.97-1.45) | (1.14-1.72) | (1.04-1.17) |
|  | **Lymphocyte Count (x 10^9^/L)** | | | | | |
|  | **0.09-1.44** | **>1.44-1.74** | **>1.74-2.03** | **>2.03-2.44** | **>2.44-6.08** | **Linear** |
|  | **(n=2149)** | **(n=2123)** | **(n=2141)** | **(n=2120)** | **(n=2128)** | **(per 1-SD* Increase)** |
| **AF Cases** | 282 | 252 | 271 | 279 | 230 | 1314 |
| **Model HR** | 1 | 0.89 | 0.90 | 0.92 | 0.72 | 0.92 |
| **(95% CI)** | (Reference) | (0.75-1.06) | (0.75-1.06) | (0.77-1.09) | (0.60-0.87) | (0.86-0.98) |
|  | **Neutrophil Count/Lymphocyte Count Ratio** | | | | | |
|  | **0.07-1.18** | **>1.18-1.58** | **1.59-2.02** | **2.03-2.56** | **2.57-53.0** | **Linear** |
|  | **(n=2140)** | **(n=2124)** | **(n=2238)** | **(n=2044)** | **(n=2115)** | **(per 1-SD* Increase)** |
| **AF Cases** | 184 | 231 | 253 | 306 | 340 | 1314 |
| **Model HR** | 1 | 1.15 | 1.13 | 1.47 | 1.49 | 1.05 |
| **(95% CI)** | (Reference) | (0.94-1.41) | (0.92-1.38) | (1.20-1.80) | (1.21-1.82) | (1.02-1.08) |
|  | **Monocyte Count (x 10^9^/L)** | | | | | |
|  | **0-0.22** | **>0.22-0.29** | **0.30-0.37** | **>0.37-0.48** | **>0.48-3.72** | **Linear** |
|  | **(n=2149)** | **(n=2116)** | **(n=2145)** | **(n=2179)** | **(n=2072)** | **(per 1-SD* Increase)** |
| **AF Cases** | 212 | 242 | 283 | 279 | 298 | 1314 |
| **Model HR** | 1 | 1.13 | 1.21 | 1.07 | 1.10 | 1.04 |
| **(95% CI)** | (Reference) | (0.94-1.36) | (1.01-1.45) | (0.89-1.28) | (0.92-1.32) | (0.99-1.10) |
|  | **Eosinophil Count (x 10^9^/L)** | | | | | |
|  | **0** | **0.01-0.09** | **>0.09-0.15** | **>0.15-0.23** | **>0.23-3.16** | **Linear** |
|  | **(n=2287)** | **(n=1983)** | **(n=2137)** | **(n=2135)** | **(n=2119)** | **(per 1-SD* Increase)** |
| **AF Cases** | 257 | 219 | 245 | 284 | 309 | 1314 |
| **Model HR** | 1 | 1.17 | 1.06 | 1.15 | 1.24 | 1.05 |
| **(95% CI)** | (Reference) | (0.96-1.42) | (0.87-1.29) | (0.95-1.40) | (1.03-1.51) | (1.00-1.10) |
|  | **Basophil Count (x 10^9^/L)** | | | | | |
|  | **0** | | **0.01-0.05** | **>0.05-0.62** | | **Linear** |
|  | **(n=5450)** | | **(n=1763)** | **(n=3448)** | | **(per 1-SD* Increase)** |
| **AF Cases** | 670 | | 169 | 475 | | 1314 |
| **Model HR** | 1 | | 0.90 | 1.01 | | 0.99 |
| **(95% CI)** | (Reference) | | (0.75-1.07) | (0.89-1.14) | | (0.94-1.05) |

**Model:** Cox proportional hazards model adjusted for age, race, sex, study site, body mass index, chronic obstructive pulmonary disease, diabetes mellitus, drinking status, educational level, height, pack-years, smoking status, systolic blood pressure, use of antihypertensive medications, prevalent heart failure, myocardial infarction, or stroke at baseline.

***Total WBC Count SD=1.70 x 10^9^/L, Neutrophil Count SD=1.42 x 10^9^/L, Lymphocyte Count SD=0.64 x 10^9^/L, Neutrophil/Lymphocyte Ratio SD=1.25, Monocyte Count SD=0.18 x 10^9^/L, Eosinophil Count SD=0.15 x 10^9^/L, Basophil Count SD=0.04 x 10^9^/L**

**Table I. Hazard ratio (HR) and 95% confidence interval (CI) of total white blood cell (WBC) count and WBC differential by atrial fibrillation (AF) ascertainment, Atherosclerosis Risk in Communities Study, 1987 to 2010**

|  | **Total WBC Count (x 10^9^/L)** | | | | |  |
| --- | --- | --- | --- | --- | --- | --- |
|  | **3.0-4.6** | **4.7-5.4** | **5.5-6.2** | **6.3-7.4** | **7.5-12.0** | **Linear** |
|  | **(n=2970)** | **(n=2966)** | **(n=2769)** | **(n=3002)** | **(n=2793)** | **(per 1-SD* Increase)** |
| **ECG AF Cases** | 15 | 23 | 25 | 29 | 24 | 116 |
| **Model HR** | 1 | 1.21 | 1.14 | 1.13 | 0.95 | 0.88 |
| **(95% CI)** | (Reference) | (0.63-2.34) | (0.59-2.20) | (0.59-2.17) | (0.47-1.92) | (0.70-1.09) |
| **AF Hospitalization Cases** | 295 | 325 | 370 | 444 | 469 | 1903 |
| **Model HR** | 1 | 1.00 | 1.08 | 1.16 | 1.24 | 1.09 |
| **(95% CI)** | (Reference) | (0.85-1.17) | (0.92-1.26) | (0.99-1.35) | (1.06-1.46) | (1.04-1.15) |
|  | **Neutrophil Count (x 10^9^/L)** | | | | |  |
|  | **0.22-2.30** | **>2.30-2.96** | **2.97-3.60** | **3.61-4.55** | **4.56-9.76** | **Linear** |
|  | **(n=2141)** | **(n=2140)** | **(n=2116)** | **(n=2132)** | **(n=2132)** | **(per 1-SD* Increase)** |
| **ECG AF Cases** | 4 | 12 | 18 | 23 | 21 | 78 |
| **Model HR** | 1 | 2.40 | 2.97 | 3.13 | 2.60 | 1.09 |
| **(95% CI)** | (Reference) | (0.75-7.66) | (0.96-9.22) | (1.02-9.64) | (0.81-8.31) | (0.85-1.41) |
| **AF Hospitalization Cases** | 182 | 223 | 246 | 279 | 361 | 1291 |
| **Model HR** | 1 | 1.15 | 1.19 | 1.27 | 1.61 | 1.16 |
| **(95% CI)** | (Reference) | (0.94-1.41) | (0.97-1.46) | (1.03-1.56) | (1.31-1.97) | (1.09-1.23) |
|  | **Lymphocyte Count (x 10^9^/L)** | | | | |  |
|  | **0.09-1.44** | **>1.44-1.74** | **>1.74-2.03** | **>2.03-2.44** | **>2.44-6.08** | **Linear** |
|  | **(n=2149)** | **(n=2123)** | **(n=2141)** | **(n=2120)** | **(n=2128)** | **(per 1-SD* Increase)** |
| **ECG AF Cases** | 19 | 21 | 15 | 16 | 7 | 78 |
| **Model HR** | 1 | 1.15 | 0.79 | 0.87 | 0.34 | 0.68 |
| **(95% CI)** | (Reference) | (0.61-2.15) | (0.40-1.56) | (0.44-1.73) | (0.14-0.84) | (0.51-0.89) |
| **AF Hospitalization Cases** | 276 | 246 | 263 | 276 | 230 | 1291 |
| **Model HR** | 1 | 0.87 | 0.88 | 0.91 | 0.71 | 0.92 |
| **(95% CI)** | (Reference) | (0.73-1.03) | (0.74-1.04) | (0.76-1.07) | (0.59-0.86) | (0.87-0.98) |
|  | **Neutrophil Count/Lymphocyte Count Ratio** | | | | |  |
|  | **0.07-1.18** | **>1.18-1.58** | **1.59-2.02** | **2.03-2.56** | **2.57-53.0** | **Linear** |
|  | **(n=2140)** | **(n=2124)** | **(n=2238)** | **(n=2044)** | **(n=2115)** | **(per 1-SD* Increase)** |
| **ECG AF Cases** | 3 | 8 | 18 | 27 | 22 | 78 |
| **Model HR** | 1 | 2.08 | 4.01 | 6.39 | 4.32 | 1.11 |
| **(95% CI)** | (Reference) | (0.53-8.15) | (1.10-14.56) | (1.81-22.64) | (1.19-15.68) | (1.00-1.24) |
| **AF Hospitalization Cases** | 182 | 229 | 250 | 295 | 335 | 1291 |
| **Model HR** | 1 | 1.22 | 1.22 | 1.55 | 1.70 | 1.07 |
| **(95% CI)** | (Reference) | (0.99-1.50) | (0.99-1.51) | (1.26-1.90) | (1.39-2.09) | (1.04-1.10) |

**Model:** Cox proportional hazards model adjusted for age, race, sex, study site, body mass index, chronic obstructive pulmonary disease, diabetes mellitus, drinking status, educational level, height, pack-years, smoking status, systolic blood pressure, use of antihypertensive medications, and prevalent heart failure, myocardial infarction, or stroke at baseline.

***Total WBC Count SD=1.70 x 10^9^/L, Neutrophil Count SD=1.42 x 10^9^/L, Lymphocyte Count SD=0.64 x 10^9^/L, Neutrophil/Lymphocyte Ratio SD=1.25**
